# The Incidence and Short-term Outcomes of Acute Respiratory Illness with Cough in Children from a Socioeconomically Disadvantaged Urban Community in Australia: A Community-Based Prospective Cohort Study

**DOI:** 10.3389/fped.2017.00228

**Published:** 2017-10-31

**Authors:** Kerry K. Hall, Anne B. Chang, Jennie Anderson, Daniel Arnold, Vikas Goyal, Melissa Dunbar, Michael Otim, Kerry-Ann F. O’Grady

**Affiliations:** ^1^Institute of Health and Biomedical Innovation, Centre for Children’s Health Research, Queensland University of Technology, South Brisbane, QLD, Australia; ^2^Department of Respiratory Medicine, Lady Cilento Children’s Hospital, South Brisbane, QLD, Australia; ^3^Menzies School of Health Research, Charles Darwin University, Tiwi, NT, Australia; ^4^Caboolture Community Medical, Caboolture, QLD, Australia; ^5^Child Health Research Centre, The University of Queensland, Brisbane, QLD, Australia; ^6^Department of Health Services Administration, College of Health Sciences, University of Sharjah, Sharjah, United Arab Emirates

**Keywords:** acute respiratory illness, cough, incidence, predictors, clinical outcomes, Aboriginal and Torres Strait Islander, cohort study

## Abstract

**Background:**

Acute respiratory illnesses with cough (ARIwC) are predominant causes of morbidity in Australian Indigenous children; however, data on disease burden in urban communities are scarce. This study aimed to determine the incidence of ARIwC, the predictors of recurrent (≥4 episodes) ARIwC, and development of chronic cough following an ARIwC in urban, predominantly Indigenous, children aged <5 years from northern Brisbane, Australia.

**Methods:**

Prospective cohort study of children aged <5 years registered with a primary healthcare center. ARIwC episodes and outcomes were collected for 12 months. Recurrent ARIwC was defined as ≥4 episodes in 12 months. Chronic cough was defined as cough lasting >4 weeks. Children who developed chronic cough were reviewed by a pediatric pulmonologist. Incidence densities per child-month of observation were calculated and predictors of recurrent ARIwC and chronic cough were evaluated in logistic regression models.

**Results:**

Between February 2013 and November 2015, 200 children were enrolled; median age of 18.1 months, range (0.7–59.7 months) and 90% identified as Indigenous. A total of 1,722 child-months of observation were analyzed (mean/child = 8.58, 95% CI 8.18–9.0). The incidence of ARIwC was 24.8/100 child-months at risk (95% CI 22.3–27.5). Twenty-one children (10.5%) experienced recurrent ARIwC. Chronic cough was identified in 70/272 (25.7%) episodes of ARIwC. Predictors of recurrent ARIwC were presence of eczema, mold in the house, parent/carer employment status, and having an Aboriginal and Torres Strait Islander mother/non-Aboriginal and Torres Strait Islander father (compared to both parents being Aboriginal and Torres Strait Islander). Predictors of chronic cough included being aged <12 months, eczema, childcare attendance, previous history of cough of >4 weeks duration, having an Aboriginal and Torres Strait Islander mother/non-Aboriginal and Torres Strait Islander father (compared to both parents being Aboriginal and Torres Strait Islander), and a low income. Of those with chronic cough reviewed by a pediatric pulmonologist, a significant underlying disorder was found in 14 children (obstructive sleep apnea = 1, bronchiectasis = 2, pneumonia = 2, asthma = 3, tracheomalacia = 6).

**Discussion:**

This community of predominantly Aboriginal and Torres Strait Islander and socially disadvantaged children bear a considerable burden of ARIwC. One in 10 children will experience more than three episodes over a 12-month period and 1 in five children will develop chronic cough post ARIwC, some with a serious underlying disorder. Further larger studies that include a broader population base are needed.

## Introduction

Acute respiratory illnesses (ARIs) are leading causes of childhood mortality and morbidity in developed and developing countries alike. Indigenous children in developed countries have high ARI hospitalization rates similar to that of children in developing countries (1–3). Cough (particularly if wet), when present, likely reflects lower airway diseases, is a key symptom of chronic lung disease (4) and represents a significant burden to families (5). Recurrent episodes of ARI, particularly lower respiratory tract infections (LRTIs) can lead to the development of chronic suppurative lung disease and bronchiectasis (6). These conditions cause substantial respiratory morbidity and mortality in developing countries (7) and vulnerable populations in developed countries (8). Thus prevention, early identification, and management of ARI with cough (ARIwC) are important public health and clinical goals.

The burden of ARI in Australia’s Aboriginal and Torres Strait Islander population is disproportionate to population size (9) and, in some regions of Australia, higher than that reported in developing countries (3). Although over half of Australia’s Aboriginal and Torres Strait Islander population live in urban or regional areas, the majority of health research is conducted in remote communities and this impacts on the ability to “close the gap” (10, 11). Studies on ARI in Aboriginal and Torres Strait Islander children have largely focused on remote communities with substantial social disadvantage. In contrast, there are no cohort studies that have comprehensively investigated ARIwC and its predictors and outcomes in urban Aboriginal and Torres Strait Islander children who can access several types of primary care services such as Aboriginal Community Controlled Health Organizations (ACCHOs), Aboriginal and Torres Strait Islander friendly general practices (GPs), mainstream GPs, and emergency departments (EDs) for acute care. Availability of such data may inform future targeted interventions. Data from Aboriginal and/or Torres Strait Islander-focused primary care, collected by an Aboriginal researcher in a setting that is culturally appropriate, are likely more reflective of factors important to Aboriginal and Torres Strait Islander people compared to other settings (e.g., from EDs).

Thus, we determined the incidence of ARIwC and the predictors of recurrent (≥4 episodes) ARIwC and chronic cough (>4 weeks duration) over a 12-month period in urban, predominantly Aboriginal and Torres Strait Islander, children aged <5 years from a socioeconomically disadvantaged community in northern Brisbane, Australia. Our specific primary objectives in this cohort of children were to (a) determine the incidence ARIwC and the recurrence of ARIwC (≥4 episodes/year) over a 12-month period and (b) determine the proportion of children who develop chronic cough (≥4 weeks duration) and their etiology following an ARIwC. Our secondary objectives were to (a) determine the demographic, clinical, environmental, socioeconomic, and cultural predictors of recurrent ARIwC and chronic cough post ARIwC and (b) describe health service utilization and medication use for ARIwC.

## Materials and Methods

### Setting

The study was conducted in a primary health care center [Caboolture Community Medical (CCM)] in an outer suburb of Brisbane, Australia. The district’s population approximates 378,045 and 2.24% are Indigenous. Since 2011 CCM has provided services to over 11,300 residents of the region; 59% are Aboriginal and Torres Strait Islander. Thus, CCM has provided at least one service to approximately 78% of the district’s Aboriginal and Torres Strait Islander population.

### Design

We undertook a prospective cohort study of children aged <5 years registered with CCM that serves a socially disadvantaged urban community with a high proportion of Aboriginal and Torres Strait Islander patients. The full study protocol has been previously published (12). Briefly, children were enrolled at the time of presentation to the clinic for any reason (including accompanying another person) and followed up monthly via phone or face-to-face contacts for 12 months.

### Participants and Recruitment

Children were eligible for inclusion if (a) they were aged <5 years at the time of enrollment; (b) parents were willing and able to complete the study requirements; and (c) the family was not intending to move from the study area in 12 months following enrollment. No exclusion criteria applied. An Aboriginal research officer undertook all recruitment and follow-up procedures. Written informed consent was obtained from parents/legal guardians following provision of a detailed information statement.

### Case Definitions

1. ARIwC was defined as an acute illness (i.e., <14-day duration) with cough as a symptom (13) and the presence of one or more other local or systemic symptoms consistent with a respiratory illness (e.g., runny nose, fever, shortness of breath, and wheeze).
2. Recurrent ARIwC was defined as ≧4 episodes of ARIwC in a 12-month period (14). A greater than 3 day and night break in cough was required to classify an episode as a new illness.
3. Chronic cough was defined a cough lasting >4 weeks (13) with no more than a 3-day break in cough in the preceding month.

### Data Collection

Data were collected at enrollment and then monthly for 12 months via detailed questionnaires administered by an Aboriginal research officer to parents/carers and review of medical records at CCM. Data collected included demographic, epidemiological, cultural, socioeconomic, and clinical data at baseline and at each monthly follow-up. At parent report of the onset of an ARIwC, a detailed illness report was completed and the child was placed on weekly follow-up, consisting of a questionnaire completed by telephone and/or face-to-face contact, for four weeks to determine cough persistence, health service utilization, and medication use during the illness. Children who had had no more than a three-day break in cough over the four weeks were classified as chronic cough at day 28 and reviewed by a pediatric pulmonologist within two weeks. Loss to follow-up was defined as two consecutive months without successful contact with the family; loss to follow-up during an ARIwC episode was defined as two consecutive weeks without contact.

### Data Analyses

The incidence density of ARIwC over a 12-month period was calculated by dividing the number of events by the person time at risk and presented as incidence per 100 child-months at risk with the corresponding 95% confidence intervals (CIs); the latter calculation assumed a Poisson distribution. The duration of ARIs (days or weeks) was subtracted from total child-months of observation to obtain child-months at risk. Incidence densities were calculated for the study cohort as a whole and by gender, age, and Indigenous status. Incidence densities by age group are presented as age at the month of observation, not by age at enrollment in order to more accurately reflect the age at which illness occurred. The proportion of children who developed recurrent ARIwC and those who developed chronic cough following ARIwC were calculated and presented with their respective 95% CIs.

Univariate analyses were performed to compare child characteristics between children who did and did not have recurrent ARIwC and children who did and did not develop chronic cough. This was undertaken for the cohort as a whole and then for Indigenous children only. Children aged 36 months and older were chosen as the reference group for outcomes by age group given the risk for ARI is highest in younger infants (15, 16). For the chronic cough analyses, only the first episode of chronic cough over the 12-month follow-up period was included in the analysis. Chi-square tests were performed on binary variables and the logistic command was used in Stata for variables with more than two categories.

To identify the predictors of chronic cough and recurrent ARIwC, we initially planned to use variables satisfying an *a priori* significance of <0.2 at a univariate level. From here a backwards, stepwise logistic regression was to be used to assess each variable individually. However, there were too many variables satisfying these criteria to validly include in modeling given the sample size, and this was the same for variables satisfying an *a priori* significance of <0.1. Hence only those variables with significance of <0.05 were included in the models.

### Ethics and Cultural Oversight

The study was approved by Human Research Ethics Committees of the Queensland Children’s Health Services (HREC/12/QRCH/169), University of Queensland (2012001395), and Queensland University of Technology (1300000741). The study was registered with the Australian New Zealand Clinical Trials Registry (ACTRN 12614001214628). Cultural oversight was provided by an Indigenous Research Reference Group. This group consisted of Aboriginal Elders, clinicians, researchers, health service providers, and community members. Community approval for the study was also obtained.

## Results

### Study Cohort

Between February 2013 and November 2015, 403 children were screened and 200 were enrolled in the study. The median age of the children enrolled was 18.1 months (range, 0.7–59.7 months); 55% were male and 90% identified as Aboriginal and Torres Strait Islander. Detailed analyses of the characteristics of the Aboriginal and Torres Strait Islander children enrolled in the study have been previously published (17). Of those not enrolled, 44 (21.6%) were ineligible, 72 (35.4%) refused, and 87 (42.8%) were not enrolled for other reasons such as homelessness or not with a primary carer/legal guardian at the time they were approached about the study and therefore not able to obtain valid consent. There were no significant differences in age and gender between those enrolled and not enrolled.

At the 12-month time point, 52 (26%) children had incomplete data and 11 parents withdrew consent at various time points. Of the children lost to follow up, there were 10 children with no follow-up data, the remaining 53 children had 172 months of observation for analysis [mean = 3.24 child-months of observation (95% CI 2.77–3.76)]. Hence there were a total of 1,722 child-months of observation available for analysis [mean = 8.58 (95% CI 8.18–9.00) child-months of observation] and 1,479 child-months at risk as the denominator for incidence densities.

### Overall Incidence of ARIwC

There were 367 reported ARIwC episodes in 200 children; median of one episode per child (range 0–8) and 45 children had none. Of those with no ARIwC episodes, the total months of observation were 244 (5.4 months per child, 95% CI 4.76–6.14) and 27 (60%) of these children were withdrawn or lost to follow-up during the study period. Of these, 20 (44%) were male, 40 (88.8%) were Aboriginal and Torres Strait Islander and 19 (42.2%) were aged less than 2 years.

The overall incidence density of ARIwC for the whole cohort was 24.9 cases per 100 child-months at risk (95% CI 22.3–27.5). The rates were 23.1 (95% CI 19.9–26.6) for males and 27.0 (95% CI 23.2–31.3) for females. Incidence density of ARIwC was 23.7 (95% CI 21.2–26.5) in the Aboriginal and Torres Strait Islander children and 34.3 (95% CI 25.7–45.3) in the non-Aboriginal and Torres Strait Islander children. The higher rates in the non-Aboriginal and Torres Strait Islander children were predominantly due to three children with multiple, prolonged illnesses, one of whom were subsequently diagnosed with bronchiectasis. The incidence densities by age group are presented in Table 1.

**Table 1:** Incidence of ARIwC over 12 months in children aged less than 5 years from an urban, disadvantaged community, by age-group for time at risk.

Age group (months)	Events	Months at risk	Incidence/100 child-months at risk	95% CI
<6	22	83.68	26.2	16.1–40.1
6 to <12	75	231.84	32.3	26.4–38.9
12 to <24	133	477.87	27.8	23.9–32.1
24 to <36	45	277.59	16.2	12.1–21.1
36 to <60	66	346.03	19.0	15.0–23.6
≧60	26	56.69	45.8	32.9–60.2
Total	367	1,473.74	24.9	22.7–27.2

### Health Service Utilization, Medication Use, and Parent Knowledge

Complete data on health service utilization and medication use were available for 330 (89.9%) episodes of ARIwC. A health professional was consulted in 210 episodes (63.6%) and of these 160 (76.2%) were GP attendances and 34 were ED presentations (16.2%). There were 25 hospitalizations for ARIwC for 15 children (hospitalization rate of 1.5 per 100 child-months of observation); two children were hospitalized on three occasions, six children were hospitalized twice, and the remainder had one each. The median length of hospital stay was 2 days (range 1–11). Four admissions resulted from specialist reviews of children with chronic cough as part of the study, and chest CT scans and bronchoscopies were performed; two children were subsequently diagnosed with bronchiectasis.

Antibiotics were prescribed in 105 (31.8%) episodes. This included 7/49 (14.3%) episodes in which the symptom was dry cough only (i.e., no fever or wet cough), 87/251 (34.7%) episodes in which wet cough was present, and 54/118 (45.8%) episodes in which both wet cough and fever were present. Oral steroids were prescribed in 16 (4.9%) episodes, inhaled corticosteroids in 9 (2.7%), bronchodilators in 22 (6.7%), and use of over-the-counter cough medicines were reported in four episodes (1.2%). Investigations were performed in 24 (7.3%) episodes.

Data on parental cough knowledge and antibiotic data were available from 152 parents (76%). Cough lasting more than 4 weeks and a wet cough were considered abnormal by 144 (95%) parents. Fifty-two parents (34.2%) thought a child should be prescribed antibiotics for cough, 63 (41.4%) did not think antibiotics should be prescribed for cough and 37 (24.3%) were undecided. One hundred twenty-nine parents (84.8%) stated that if your child was prescribed antibiotics that they should finish the course, 20 (13.1%) said they should be taken until no symptoms were present.

### Recurrent ARIwC

Twenty-one children (10.5%) experienced recurrent ARIwC during the follow-up period. Of these, 13 (61.9%) were male, 16 (76.2%) were Aboriginal and Torres Strait Islander and 15 (71.4%) were aged <2 years (Table 2). Of the 21 children identified with recurrent ARIwC, there were 105 illnesses recorded, with a mean of 5 (95% CI 4.0–6.0) illnesses per child, and a mean of 7.5 (95% CI 6.3–8.7) health care visits over the observation period. There were 158 health professional consultations; 124 (78.4%) were GP visits, 17 (10.7%) ED visits, and eight hospitalizations in eight children. Of the children with recurrent ARIwC, antibiotics were prescribed in 35% of all illnesses.

**Table 2:** Predictors of recurrent acute respiratory illness with cough in children aged less than 5 years from a disadvantaged urban community.

All children (*n* = 191)	aRR	95% CI	*P* value	Indigenous children only (*n* = 174)	aRR	95% CI	*P* value
Male gender	1.37	0.44–4.23	0.98	Male gender	1.16	0.33–4.00	0.92
**Age group in months**				**Age group in months**			
36 to >60			Ref	36 to >60			Ref
24 to <36 months	1.29	0.17–9.47	0.051	24 to <36 months	1.72	0.23–12.8	0.35
12 to <24 months	2.15	0.37–12.34	0.24	12 to <24 months	2.01	0.34–11.8	0.22
6 to <12 months	1.70	0.30–9.72	0.66	6 to <12 months	1.55	0.26–9.22	0.51
<6 months	1.15	0.20–6.46	0.49	<6 months	0.85	0.12–5.70	0.71
Eczema ever	13.10	2.78–61.5	0.001	Eczema ever	12.6	2.69–59.5	0.002
Mold in house	0.07	0.012–0.44	0.005	Mold in house	0.06	0.007–0.62	0.01
**Parent Indigenous status**				**Parent Indigenous status**			
Both parents Indigenous			Ref	Both parents Indigenous			Ref
Indigenous father/non-Indigenous mother	3.60	0.42–30.85	0.16	Indigenous father/non-Indigenous mother	3.16	0.39–25.1	0.25
Indigenous mother/non-Indigenous father	10.42	1.27–85.39	0.03	Indigenous mother/non-Indigenous father	8.94	1.16–68.4	0.07
Both parents non-Indigenous^a^				Both parents non-Indigenous	NA	NA	NA
**Mothers employment status**				**Mothers employment status**			
Full time			Ref	Full time^a^			Ref
Casual/part time	0.12	0.01–1.23	0.04	Casual/part time	0.0.28	0.02–2.83	0.19
Unemployed	0.07	0.01–0.38	0.004	Unemployed	0.09	0.01–0.56	0.01

*^a^Omitted due to collinearity*.

Based on the univariate analyses presented in Tables S1–S4 in Supplementary Material, a logistic regression model was constructed to evaluate the association between child, parental, household, and environmental characteristics and recurrent ARIwC. These were performed firstly including all children and then for Aboriginal and Torres Strait Islander children only; the latter model included cultural characteristics. Age and gender were retained irrespective of final statistical significance. The model results are presented in Table 2. We found that a history of eczema, having mold in the house, households in which only the mother identified as Aboriginal and Torres Strait Islander (compared to both parents identifying as such), and the employment status of the mother were associated with recurrent ARIwC.

### Duration of Cough and Occurrence of Chronic Cough

Of the 367 ARIwC episodes, weekly follow-up data over a 4-week period were available for 272 episodes in 147 children. Lack of follow-up data was a result of some ARIwC being identified too late to validly commence follow-up from the reported onset date. Failure to contact the parent/guardian at any timepoint over the 4-weeks led to the outcome of chronic cough being unknown. If it was known the cough had stopped prior to LTFU, the presence of persistent cough (i.e., no break in cough during the 28-day follow-up period) at all subsequent timepoints was classified as “no.” The presence of cough at each weekly timepoint together with cough type is presented in Table 3.

**Table 3:** Weekly cough persistence^a^ and cough type over 28 days in 272 episodes of ARIwC.

*N* = 272 episodes	Cough has not stopped	Cough type	Cough has stopped	Unknown
Day 7	194 (71.3)	Wet 49 (25.6) Dry 47 (24.2) Variable 81 (41.8) N/A* 17 (8.8)	40 (14.7)	38 (14.0)
Day 14	150 (55.2)	Wet 36 (24.0) Dry 42 (28.0) Variable 45 (30.0) N/A 27 (18.0)	78 (28.7)	44 (16.2)
Day 21	111 (40.8)	Wet 25 (22.5) Dry 31 (27.9) Variable 36 (32.4)	114 (41.9)	47 (17.4)
Day 28	70 (25.7)	Wet 18 (25.7) Dry 20 (28.6) Variable 26 (37.1) N/A 6 (25.7)	152 (55.9)	50 (18.4)

*^a^There has been no break in cough of >3 days in the week prior to each contact timepoint. At each weekly contact parents were asked whether the child’s cough had stopped for >3 days in the preceding week, as any break in cough of that duration at any time during the 28 days of follow-up meant the child did not meet the study definition of chronic cough at day 28*.

*N/A, Not applicable.

Chronic cough at the day-28 follow-up was identified in 70/272 (25.7%) episodes in 43/147 (29.2%) children with follow-up or 21.5% (43/200) of the entire study cohort; cough status was unknown at day-28 in 59 (21.7%) episodes for 53 children. Prior to their first episode of chronic cough identified in the study, 53.4% (23/43) children had no prior ARIwC reported in the period of observation before the chronic cough episode, 11 children had one prior episode, seven had two, one had three, and one child had six episodes. Seventeen children had one episode of chronic cough only; 14 children had 2, seven had three episodes and three children had four episodes over the follow-up period. Cough status and type at each time-point of follow-up for the 272 episodes are presented in Table 3.

The baseline characteristics of children who did and did not have an episode of chronic cough are presented in Tables S5–S8 in Supplementary Material. Based on the univariate analyses, a logistic regression model was constructed to evaluate the association between child, parental, household and environmental characteristics, and parental knowledge with the development of chronic cough. These were performed first including all children and then for Indigenous children only; the latter model included cultural characteristics. Age and gender were retained irrespective of final statistical significance. The model results are presented in Table 4.

**Table 4:** Predictors of chronic cough following ARIwC in children aged less than 5 years from an urban disadvantaged community.

All children (*n* = 194)	aRR	95% CI	*P* value	Indigenous Children only (*n* = 176)	aRR	95% CI	*P* value
Male gender	0.77	0.34–1.75	0.53	Male gender	0.51	0.21–1.26	0.14
**Age group in months**				**Age group in months**			
36 to >60			Ref	36 to >60			Ref
<24 to <36 months	0.33	0.05–2.11	0.24	<24 to <36 months	0.38	0.05–2.49	0.31
>12 to <24 months	2.83	0.81–9.91	0.10	>12 to <24 months	2.43	0.66–8.85	0.17
6 to <12 months	7.32	1.62–33.0	0.01	6 to <12 months	6.41	1.32–31.0	0.02
<6 months	4.74	1.12–20.0	0.03	<6 months	4.39	0.98–19.5	0.05
Eczema ever	3.76	1.14–12.3	0.02	Eczema ever	3.99	1.21–13.1	0.02
Childcare attendance	3.56	1.28–9.92	0.01	Childcare attendance	3.04	1.02–9.06	0.04
Previous chronic cough	3.39	1.29–8.89	0.01	Previous chronic cough	2.45	0.88–6.82	0.10
**Parent Indigenous status**				**Parent Indigenous status**			
Both parents Indigenous			Ref	Both parents Indigenous			Ref
Indigenous father/non-Indigenous mother	3.06	0.898–10.4	0.06	Indigenous father/non-Indigenous mother	2.73	0.81–9.11	0.09
Indigenous mother/non-Indigenous father	6.23	1.82–21.2	0.003	Indigenous mother/non-Indigenous father	5.41	1.63–17.9	0.005
Both parents non-Indigenous^a^				Both parents non-Indigenous	NA	NA	NA
**Household income**				**Household income**			
$52,000 to <$78,000	0.46	0.098–2.22	0.33	$52,000 to <$78,000	0.30	0.06–1.57	0.14
$26, 000 to <$52,000	0.19	0.050–0.759	0.01	$26, 000 to <$52,000	0.18	0.04–0.73	0.01
<$26,000	0.43	0.123–1.56	0.18	<$26,000	0.35	0.09–1.33	0.11

*^a^Omitted due to collinearity*.

### Diagnostic Outcomes of Children with Chronic Cough

Of the 43 children who developed at least one episode of chronic cough, 26 (60.4%) were reviewed by a pediatric respiratory physician who used a standardized approach and all had a spirometry (when able) and CXR as per guidelines (18). The remainder of the children (*n* = 17) were not reviewed as parents declined to attend as the child’s cough had resolved by time of review appointment (*n* = 2) or did not attend for other reasons (*n* = 15).

Of the 26 children reviewed, 14 (53.8%) children had an underlying chronic lung disease diagnosed for the first time as direct result of the study. Among the primary diagnoses, protracted bacterial bronchitis (PBB) was the most common diagnosis *n* = 19 (73%) (Table 5). Nineteen (73.0%) children who presented for review had more than one diagnosis (Table 5). Of the three children with either asthma or bronchiectasis, all had recurrent ARIwC.

**Table 5:** Final and secondary diagnoses of 26 children with chronic cough who presented for specialist review.

Final primary diagnosis	Secondary diagnoses
Asthma, *n* = 1	Allergies/hay fever, *n* = 1
Pneumonia, *n* = 2	Obstructive sleep apnea, *n* = 1 Recurrent childhood cough, *n* = 1 Otitis media, *n* = 1

Protracted bacterial bronchitis, *n* = 19	Allergic rhinitis, *n* = 3 Eczema, *n* = 1 Pharyngomalacia, *n* = 1 Obstructive sleep apnea, *n* = 3 Tracheomalacia, *n* = 5 Allergic Rhinitis, *n* = 3 Bilateral Glue Ear, *n* = 1 Failure to Thrive, *n* = 1 Tonsillitis, *n* = 3 Excess environmental tobacco smoke exposure, *n* = 1

Non-specific cough, *n* = 2	Nil

Bronchiectasis, *n* = 2	Tracheomalacia, *n* = 1 Excess environmental tobacco smoke exposure, *n* = 1

## Discussion

This is one of the few studies to investigate ARIwC incidence at a community level in Australia. Our study represents children from predominantly low socioeconomic backgrounds, with high levels of environmental tobacco smoke (ETS) exposure and 90% of families are welfare recipients (17). We focused on ARIwC rather than any ARI alone as cough is the most common reason for presentation to GPs in Australia (19); more likely associated with lower airway diseases (particularly if wet); a key symptom in chronic lung disease (4) and represents a significant burden to families (5), including Aboriginal and Torres Strait Islander families (20).

Overall one in five children will experience an ARIwC each month; one in 10 will have four or more episodes per year and one in five will have at least one episode of chronic cough. A history of eczema, having an Aboriginal and Torres Strait Islander mother/non-Aboriginal and Torres Strait Islander father (compared to both parents identifying as Aboriginal and Torres Strait Islander), and mother not in paid employment were independently associated with development of recurrent ARIwC. Health service utilization for ARIwC is high, as is the use of antibiotics. Variables independently associated with the development of chronic cough were being aged <12 months, eczema, childcare attendance, previous history of cough >4 weeks duration, and having Aboriginal and Torres Strait Islander mother/non-Aboriginal and Torres Strait Islander father (compared to both parents identifying as Aboriginal and Torres Strait Islander); having a low income was protective. A significant new underlying chronic lung disease was diagnosed in 14 children. PBB was the most common cause of chronic cough, diagnosed in 73% of children reviewed by the specialist. The lack of association with ETS, an unusual finding in studies of ARI, is likely due to the very high prevalence of smoking in the study cohort.

### ARIwC: Incidence and Risk Factors

Currently, the majority of information on ARI incidence is based on hospitalizations and ED presentations, with most studies focusing on LRTI and hospitalization rates (21). There are no urban studies that have a predominantly Aboriginal and Torres Strait Islander cohort.

Sarna and colleagues’ (22) Brisbane-based study of 154 infants followed from birth until 24 months of age reported an incidence of any ARI of 0.56/child-month, with URTI and LRTI accounting for 83% and 17% of ARI episodes per month (mean incidence of 0.47/month and 0.10/month, respectively). These rates differed from ours (average of 0.29 per child-month). This is not surprising as, although they utilized daily cards (22), cough was not a prerequisite in either definition of URTI or LRTI. However, if reported, a dry cough was considered an URTI and moist cough an LRTI. In our study, wet cough predominated suggesting that the incidence of LRTI was higher if we used the same definition. Sarna and colleagues (22) also reported that GP consultations occurred in 47.6% of episodes and antibiotics were prescribed in 21.9% of ARI episodes, lower than in our study. A Perth study (21) (1996–1998) followed 263 infants from birth until 5 years of age and utilized daily symptom diary and fortnightly phone calls. Children aged <2 years averaged four episodes of ARI/year, reducing to 2–3 episodes/year between ages 2 and 5 years (21). Forty-five percent resulted in GP visits and antibiotics were prescribed in 23.6%, similar to the Brisbane cohort (22). In the Perth study (21), ARI episodes with runny/blocked nose or dry cough were considered an URTI and episodes associated with wheeze, or cough (not further defined) and “rattly” chest were considered LRTI. To meet inclusion, infants had to be at high risk of atopy (at least one parent had doctor diagnosed atopy). A cohort study in healthy children conducted in Melbourne between July 2001 and December 2001 followed 121 children aged 12–71 months for 12 weeks utilizing a parent-completed daily symptom diary to identify episodes of influenza-like illness (ILI) (15). The authors reported an incidence of 0.53 episodes/child-month, 46.7% resulted in GP visits and antibiotics were prescribed in 17.5%. The difference between these three urban Australian studies and ours reflect not only differences in design and case definitions but marked differences in socioeconomic status. The cohorts were comprised of predominantly first-born children, high-income families, high use of childcare, low levels of ETS exposure, and small numbers of Aboriginal and Torres Strait Islander children.

Aboriginal and/or Torres Strait Islander children have higher rates of ED presentation and hospitalizations for ARI than non-Aboriginal and Torres Strait islander children (16, 23). Despite this, little is known about the community incidence of ARI in this population. A Northern Territory (NT) study reviewed clinic presentations for Aboriginal infants during the first year of life in five remote communities (24). The median number of presentations per child for any reason was 21 (IQR 15–29), with the most common reasons being URTI [median six visits per child (IQR 3–10)] and LRTI [median of three visits per children for LRTI (IQR 2–5)] (24). Another NT retrospective review of clinic records in two remote communities of children reported a median of 16 (IQR 10–22) presentations per child per year. The most common reasons for presentation were URTI (32%) and LRTI (10.7%) (25). Direct comparisons between these NT studies and ours are not possible given the different demographic and geographical profiles of the communities involved, differences in study design and differences in access to health care (single clinics in NT communities versus multiple health care services for urban children).

### Recurrent ARIwC: Rates and Risk Factors

Longitudinal studies have shown that recurrent respiratory infections in early childhood can be associated with long-term poorer respiratory health (e.g., COPD) including impaired lung function (26, 27). Despite this, the burden of recurrent ARI in childhood is unclear particularly for ARIwC. Of 21 children identified in our study with recurrent ARIwC, 105 illnesses were recorded (mean of five episodes per child, 95% CI 4.0–6.0). There were 158 health professional consultations; of these 124 (78.4%) were GP visits, 17 (10.7%) ED visits, seven (4.4%) respiratory specialist reviews, and eight (5.0%) hospitalizations. Among children with recurrent ARIwC, antibiotics were prescribed in 35% of all illnesses. Eczema, having an Aboriginal and Torres Strait Islander mother/non-Aboriginal and Torres Strait Islander father and having a mother not in paid employment were independently associated with recurrent ARIwC in our cohort with the latter being protective. The high rate of recurrent ARIwC in non-Aboriginal and Torres Strait Islander children, despite the small numbers of those children enrolled, reflects the experience of one non-Aboriginal child who was diagnosed with bronchiectasis during the study.

There are few comparable studies on recurrent ARIwC. A Finnish (28) cross-sectional analysis of data from an intervention study in children aged 1–6 years attending day care centers used the same classification of recurrent ARI as ours but did not require cough in the case definition. They reported that 44% of children aged 1–3 years had recurrent ARI in the preceding 12 months. Risk factors were mother’s education, parental history of atopy, and day care attendance while having older siblings, and furry pets reduced the risk of recurrent ARI (28). In another prospective birth cohort study, with children followed to 2 years of age (14), that defined recurrent ARI as >98 respiratory illness days per year, 10% of children experienced recurrent ARI; similar to our study but again did not require cough. The median number of illnesses in children with recurrent ARI was 9.6 (IQR 7.6–11.1) episodes per year. The only risk factor for recurrent ARI was having older siblings (adjusted OR 3.03, 95% CI 1.94–4.74); however, the factors they examined were limited in comparison to our study. Due to several differences between this study and ours, any direct comparison is problematic.

Our finding of an increased risk of recurrent ARIwC in those with atopy is not surprising as the association is well documented (29, 30). Having an Aboriginal and Torres Strait Islander mother/non-Aboriginal and Torres Strait Islander father increased the risk of recurrent ARIwC compared to both parents identifying as Aboriginal and Torres Strait Islander; the reverse combination was trending toward significance. However, this finding is likely confounded as further examination of the data indicated that those parent structures were more common in single-parent households than dual-parent households. Hence the increased risk of ARIwC may be a factor of parent structures rather than Aboriginal and Torres Strait Islander status *per se*.

Our findings of having an unemployed mother and mold present in the house as being protective factors for ARIwC are difficult to explain. We had low levels of childcare use, a known risk for ARI (31–33), in the study children and this may be related to the employment status of mothers. Our measure of mold was parent-reported and not evaluated objectively. Mold has been associated as being a risk factor for ARI in several studies (34, 35) and it is unclear why we have a reverse association. It could also be related to the amount of time children spend inside the house and use (or not) of air-conditioning; however, we did not collect those data.

### Post ARIwC: Occurrence of Chronic Cough and Risk Factors

As there are currently no published data on the occurrence and etiologies of chronic cough post ARI in Aboriginal and Torres Strait Islander children in an urban setting, we examined these factors. This is important in the context that chronic respiratory illnesses, most manifested by chronic cough, are prevalent among this population group (36). Early recognition and appropriate management may prevent the development of CSLD and bronchiectasis (37). We identified chronic cough in 70/272 (25.7%) of episodes with completed weekly follow-up and recurrent episodes were not uncommon.

There are few community-based studies of the duration and outcome of ARIwC in children. A British prospective cohort study in preschool children presenting to eight GPs with cough ≤28 days duration at time of enrollment reported that 90% children had stopped coughing at 25 days (38). These findings were supported by a systematic review of 10 studies (39). However, none of the studies involved examining the children at the point of chronic cough occurrence. Our previous prospective cohort study of 879 children aged <15 years presenting to a Brisbane tertiary pediatric ED with ARIwC identified that 20% (95% CI 17–23) of children had a persistent cough at day 28 (40); that study utilized the same methods.

We identified eczema, young age, childcare attendance, having a previous episode of chronic cough and having an Aboriginal and Torres Strait Islander mother/non-Aboriginal and Torres Strait Islander father as being associated with increased risk of developing chronic cough. Atopy, young age, and childcare attendance are well-documented risk factors for ARI, including recurrent episodes as described above (29, 30, 32, 33, 38). The association with Indigenous status of the parent is again likely to be the same as for recurrent ARIwC described above. This association suggests that assessment of parent support in the home should be an important component in the management of children with recurrent ARIwC and those with chronic cough.

### Post ARIwC: Etiologies of the Children’s Chronic Cough

On clinical review using a standardized pathway, we found that 14 children who had chronic cough had a previously undiagnosed chronic underlying lung disease. This included two children with bronchiectasis (prevalence of 7.7% of cohort reviewed by a physician). Both children had long histories of recurrent episodes of wet cough that had not been investigated. This prevalence of 8% is similar to that found (9%) in a national multicenter study involving 346 children presenting for the first time to a pediatric respiratory physician (20). One major difference in the national study was that the median duration of cough was 16 weeks (IQR 8–32) at the time of enrollment. In the aforementioned study (20), Aboriginal and Torres Strait Islander children (10 of 34) were significantly (*P* = 0.001) more likely to have bronchiectasis than non-Aboriginal and Torres Strait Islander children (21 of 312). The prevalence of newly diagnosed bronchiectasis in our ED study described above was 3.4% (4/117), none of whom were of Aboriginal and Torres Strait Islander origin (41).

Thus, given the implications of earlier diagnosis and subsequent management of children with an underlying lung disease (e.g., bronchiectasis), the review of children with persistent cough four weeks following an ARIwC at the transitional stage from sub-acute to chronic cough is warranted, particularly in those with known risk factors or recurrent ARIwC. This review should follow evidence-based guidelines (42) for the management of cough in children with early referral to tertiary care if the cough is not resolving.

### Parental Knowledge of Cough and Antibiotic Use

Parent knowledge of symptoms, illness duration, and medication use is important for clinicians to understand. This enables tailored advice and assistance with the management of the child’s illness. As far as we are aware, this is the first study to report parent knowledge of cough and antibiotic use in Aboriginal and Torres Strait Islander families. We found a high proportion of parents knew that a cough lasting more than 4 weeks and wet cough were abnormal (94.7 and 93.4%). However, a high proportion thought antibiotics should be given for cough (34%) or were unsure (24.3%) and 13% thought antibiotics should be stopped when symptoms had resolved rather than completing the full course.

It is encouraging that most parents could recognize abnormal cough and, while this may differ in other settings and/or in the parents of children who were not enrolled, it may partially explain the high health service use for ARIwC in our study. There appears to be a dearth of literature specifically addressing parent knowledge of chronic cough and wet cough. A qualitative study involving 60 parents of children aged 5 months to 17 years from a range of socioeconomic backgrounds found that a wide range of perceptions will initiate parents seeking help for a child with cough that were similar across socioeconomic groups (43). These perceptions were influenced by parent experiences with ARI (particularly other children with ARI illnesses) and their confidence and efficacy in managing illness (43).

The use of antibiotics in community managed ARI in children is an important problem globally. A systematic review of qualitative studies of parent consulting and clinician antibiotic prescribing decisions in pediatric ARI reported important influences were the perception of a threat or uncertainty with respect to the child’s illness and illness outcomes (44). They concluded that interventions aimed at improving both parent and clinician management of illness need to “fit” with social norms and ensure concerns of safety are addressed. A systematic review of interventions to influence consulting and antibiotic use for ARI in children reported that using materials that engaged the child, as well as the parent, modified parental knowledge and behavior thus reducing consultation rates (45).

### Strengths and Limitations

The strengths of this study are the comprehensiveness of the data collected and that Aboriginal researchers undertook all study procedures. The clinic was actively engaged in study design and implementation and was supported by the members of our Indigenous Research Reference Group ensuring the cultural applicability of the study. The relationships built between researchers and study parents were important in keeping families engaged during the study. These relationships allowed the parents to be heard when their child presented with ARI, especially given cough is at times considered trivial to some health professionals (4, 46). The longitudinal design with intensiveness of follow-up when ARIwC was reported allowed a comprehensive assessment of the outcomes of ARIwC in these children. The review of children with persistent cough by a pediatric respiratory physician in accordance with cough management guidelines (42) enabled the identification of underlying lung disease that would not otherwise have been detected. Our focus on ARIwC rather than any ARI provides a more specific measure of a type of ARI that is likely to be of greater burden to families.

Our study had several limitations. Firstly, being a single center study limits the generalizability of the study findings to other urban communities. However, CCM has provided a service to over 70% of the Moreton Bay’s Aboriginal and Torres Strait Islander population, despite five other Aboriginal Community Controlled Health Organizations in the region as well as two hospitals and multiple mainstream GPs. Our findings are within the ranges of incidence and health service use of other community-based studies as is the prevalence of recurrent ARIwC and chronic cough post ARIwC.

Second, we identified a large numbers of predictors associated with ARIwC in our study, however, due to small numbers in some of the variables we were unable to include them in the analysis models to further explore their relationship with study outcomes. The number of non-Aboriginal and Torres Strait Islander children in our study was small 20 (10%), and they had similar socioeconomic and social disadvantage as the Aboriginal and Torres Strait Islander children, which may explain the high rates of ARIwC in the former group. Further, larger studies are needed to confirm whether the disease burden does indeed differ between groups within the region.

Third, there are likely differences between children who were and were not enrolled; parents of children who experience frequent ARI episodes may be more inclined to enroll their child and remain in the study introducing selection bias. The extent to which this has influenced our study findings is unknown at this stage.

Also, there is also the potential of bias in our outcome estimates derived from loss-to-follow-up, particularly as those who complete the study may differ from those who do not. Loss to follow up during the weekly follow up could reflect that the child had stopped coughing or child had recovered and the parent decided that the cough was no longer of concern. In the ED cough study, loss to follow-up was associated with a milder illness and in our study, incomplete weekly follow-up data were also associated with parents not continuing after the child had stopped coughing. Missing data at monthly time points reflected parents who either withdrew from the study or had more than one missed contact attempt. However, that our overall loss to follow-up was 31.5% of children by the end of 12 months. We consider a retention rate of 68.5% as relatively successful given the characteristics of the study population (17) and the challenges associated with retaining those families in a longitudinal study. Of note is that a particular period during the study in which loss-to-follow-up was highly coincided with a 3-month period in which the Aboriginal staff were not available due to illness and other personal matters. Retention was high when they returned to duty. This highlights the importance of Aboriginal and Torres Strait Islander people being a major part of studies in this population. The resources available for the study also limited the capacity to implement measures that would have improved participant retention. Home visiting would likely have facilitated ongoing engagement of families and hence further studies should consider including sufficient resources to make this possible.

Lastly, although we restricted recruitment of children to those aged <5 years, this is not a birth cohort study. Thus, we are unable to evaluate precursors to the development of recurrent ARIwC and chronic lung disease that have occurred pre- and post-birth, particularly the changes in those exposures over time as children age.

### Possible Future Intervention Points

The etiology and development of recurrent ARIwC and chronic lung disease are complex and interventions beyond a biomedical focus are required over the long term, particularly those that address socioeconomic disadvantage. Based on our study’s findings, possible intervention points to improve respiratory health include the transitional phase from acute to chronic cough, the point at which recurrent episodes within a 12-month period are identified and, generally, enhancing the uptake and impact of the available public health interventions.

There is currently limited evidence for interventions to prevent recurrent ARI and exacerbations of chronic lung disease in children (47). Hence focusing on strategies to minimize the risk of disease and optimize early recognition and management of chronic cough are likely important, as is ongoing, high-quality research. There is good evidence that cough management pathways in children with chronic cough are effective in improving clinical outcomes (48). An algorithm previously successfully evaluated in children with chronic cough (20) is now the subject of a clinical trial in children at the transitional phase from acute to chronic cough, including children from this study’s community (49). Given recurrent ARIs in early childhood, particularly LRTIs, are known precursors to chronic lung disease, further investigation and monitoring of children when they reach the “recurrent” threshold may be warranted. At the primary care level, clinicians need ongoing education with respect to the early recognition and management of persistent cough and recurrent ARIwC and parents need to be provided with sufficient information to know when to seek care. Clinical management needs to be coupled with preventative measures such as supporting the uptake and timeliness of maternal and childhood immunizations through mechanisms that account for socioeconomic disadvantage and marginalization; exploring innovative methods to reduce environmental tobacco smoke exposure in populations that current programs do not appear to be reaching; and wider access to early childhood support programs for parents, particularly single parent families and the unemployed.

## Conclusion

This study is the most comprehensive assessment of ARIwC at the community level in a predominantly Aboriginal and Torres Strait Islander cohort of young children. Our estimated disease incidence over a 12-month period of 24.8 cases per 100 child-months and prevalence of the occurrence of chronic cough (21%) were relatively high indicating the considerable burden of disease in this community. Further, the common finding (32.6%) of an underlying chronic lung disease in these children who would otherwise not have been identified suggests that children with chronic cough should be comprehensively followed up.

## Availability of Data and Material

Study data and materials may be made available on request with appropriate human research ethics committee approval and with the consent of the participating community as required by Australian criteria for research with Indigenous communities.

## Ethics Statement

The study was approved by Human Research Ethics Committees of the Queensland Children’s Health Services (HREC/12/QRCH/169), University of Queensland (2012001395) and Queensland University of Technology (1300000741). The study was registered with the Australian New Zealand Clinical Trials Registry (ACTRN 12614001214628). Cultural oversight was provided by an Indigenous Research Reference Group. This group consisted of Aboriginal Elders, clinicians, researchers, health service providers, and community members. Community approval for the study was also obtained.

## Author Contributions

KH contributed to study design, had primary responsibility for recruitment and data collection, and wrote the first draft of the manuscript. AC contributed to study design and implementation and provided significant input to the drafting of the manuscript. JA contributed to study design, managed the study at CCM and contributed to the manuscript. DA assisted with data management and analysis and contributed to the manuscript. VG undertook respiratory physician reviews, contributed to the design of data collection at these reviews and to the final manuscript. MD assisted with recruitment, data collection, and participant follow-up. MO contributed to study design and the final manuscript. KO conceptualized the study, had overall responsibility for study conduct and completed and contributed to the manuscript. All authors approved the final manuscript.

## Conflict of Interest Statement

JA is the Director of the clinic in which this study was conducted. She had no role in the recruitment and consent of participants and did not receive financial support for the study.
