# Supplementary material for: The Incidence and Short-term Outcomes of Acute Respiratory Illness with Cough in Children from a Socioeconomically Disadvantaged Urban Community in Australia: A Community-Based Prospective Cohort Study
Source: Front Pediatr. 2017 Oct 31;5:228. doi: 10.3389/fped.2017.00228 (PMC5674932; doi:10.3389/fped.2017.00228)
Supplement: Supplementary file 1 [file Table_1.DOCX]

**Supplementary Table 1. Child characteristics of children who developed recurrent ARIw**

|  |  | All children  N=200 (%) | Recurrent ARI No  n = 179 | Recurrent ARI Yes  n = 21 (%) | ***P*** value |
| --- | --- | --- | --- | --- | --- |
| **Gender** | Female | 95 (47.5) | 87 (91.6) | 8 (8.4) | Ref |
|  | Male | 105 (52.5) | 92( 87.6) | 13 (12.4) | 0.36 |
| **Indigenous Status** | Non-Indigenous | 20 (10.0) | 15 (75.0) | 5 (25.0) | Ref |
|  | Indigenous | 180 (90.0) | 164 (91.1) | 16 (8.9) | 0.04 |
| **Age group months** | 36 ->60* | 46 (23.0) | 43 (93.5) | 3 (9.4) | Ref |
|  | <24 - <36 | 32 (16.0) | 29 (90.6) | 3 (14.2) | 0.64 |
|  | > 12 - <24 | 55 (27.5) | 48 (87.3) | 7 (12.7) | 0.30 |
|  | 6 -<12 | 60 (33.5) | 27 (90.0) | 3 (10.0) | 0.58 |
|  | < 6 | 37 | 32 (86.5) | 5(13.5) | 0.29 |
| **Birth Weight (grams)** | ≥2500 | 161 (80.5) | 143 (88.8) | 18 (11.2) | Ref |
|  | ≤ 2500 | 39 (19.5) | 36 (92.3) | 3(7.7) | 0.52 |
| **Gestational age weeks** | ≥ 37 | 159 (79.5) | 145 (91.2) | 14 (8.8) | Ref |
|  | < 37 | 41 (20.5) | 34 (82.9) | 7 (17.1) | 0.61 |
| **History of wheeze past 12 months** | No | 123 (62.1) | 111(90.2) | 12 (9.8) | Ref |
|  | Yes | 75 (37.9) | 66 (88.0) | 9 (12.0) | 0.61 |
|  | Missing | 2 | 2 | 0 |  |
| **History of Eczema ever** | No | 174 (87.8) | 159 (91.4) | 15 (8.6) | Ref |
|  | Yes | 24 (12.2) | 18 (75.0) | 6 (25.0) | 0.01 |
|  | Missing | 2 | 2 | 0 |  |
| **Hospitalised for ARI 12 months prior to enrolment** | No | 173 (87.3) | 157 (90.7) | 16 (9.25) | Ref |
|  | Yes | 25 (12.7) | 20 (80.0) | 5 (20.0) | 0.10 |
|  | Missing | 2 | 2 | 0 |  |
| **Cough> 4 weeks duration 12 months prior to enrolment** | No | 151 (76.2) | 136 (90.1) | 15 (9.9) | Ref |
|  | Yes | 47 (23.7) | 41 (87.2) | 6 (12.8) | 0.58 |
|  | Missing | 2 | 2 | 0 |  |
| **Respiratory Diagnosis 12 months prior to enrolment** | No | 130 (65.7) | 119 (91.5) | 11 (8.5) | Ref |
|  | Yes | 68 (34.3) | 58 (85.3) | 10 (14.7) | 0.35 |
|  | Missing | 2 | 2 | 0 |  |
| **Season enrolled** | Summer | 57 (28.5) | 53 (92.9) | 4 (7.1) | Ref |
|  | Autumn | 53 (26.5) | 50 (94.3) | 3 (5.7) | 0.77 |
|  | Winter | 37 (18.5) | 32 (86.5) | 5 (13.5) | 0.30 |
|  | Spring | 53 (26.5) | 44 (83.1) | 9 (1630) | 0.11 |

**Supplementary Table 2. Parental characteristics of children with recurrent ARIwC**

|  |  | All children  N = 200(%) | Recurrent ARI No  n = 179 (%) | Recurrent ARI Yes  n =21 (%) | P  Value |
| --- | --- | --- | --- | --- | --- |
| **Maternal age at birth of child** | 40 + years | 5 (2.5) | 4 (80.0) | 1 (20.0) | Ref |
|  | 35 - <40 years | 19 (9.6) | 16 (84.2) | 3 (15.8) | 0.82 |
|  | 30 - <35 years | 31 (15.7) | 27 (87.1) | 4 (12.9) | 0.67 |
|  | 25- <30 years | 41 (20.7) | 37 (90.2) | 4 (9.8) | 0.49 |
|  | <25 years | 102 (51.5) | 93 (91.2) | 9 ( 8.8) | 0.41 |
|  | Missing | 2 | 2 |  |  |
| **Paternal age at birth of child** | 40 + years | 21 (10.7) | 18 (85.7) | 3 (14.3) | Ref |
|  | 35 - <40 years | 28 (14.2) | 26 (92.9) | 2 (7.1) | 0.42 |
|  | 30 - <35 years | 25 (12.7) | 21 (84.0) | 4 (16.0) | 0.87 |
|  | 25- <30 years | 53 (26.9) | 44 (83.0) | 9(17.0) | 0.77 |
|  | <25 years | 70 (35.5) | 67 (95.7) | 3 (4.3) | 0.12 |
|  | Missing | 3 | 3 |  |  |
| **Indigenous Parent status** | Both Indigenous | 59 (30.0) | 57 (96.6) | 2 (3.4) | Ref |
|  | Indigenous dad/ non-Indigenous mum | 59 (30.0) | 54 (91.5) | 5 (8.5) | 0.25 |
|  | Indigenous mum/ non-Indigenous dad | 59 (30.0) | 50 (84.7) | 9(15.3) | 0.04 |
|  | Both non-Indigenous | 20(10.0) | 15 (75.0) | 5 (25.0) | 0.01 |
| ***Father* education** | Tertiary degree | 2 (1.0) | 1 (50.0) | 1 (50.0) | Ref |
|  | Secondary school/ trade | 82 (41.9) | 70 (85.3) | 12 (14.7) | 0.22 |
|  | Did not complete high school | 82 (41.9) | 76 (92.7) | 6 (7.3) | 0.08 |
|  | Unknown | 30 (15.2) | 28 (93.3) | 2 (6.7) | 0.09 |
|  | Missing | 4 | 2 | 0 |  |
| ***Mother* education** | Tertiary degree | 10 (5.0) | 7 (70.0) | 3 (30.0) | Ref |
|  | Secondary school/ trade | 108 (54.8) | 93 (86.1) | 15 (13.9) | 0.18 |
|  | Did not complete high school | 77 (39.1) | 74 (96.1) | 3 (3.9) | 0.09 |
|  | Unknown | 2 (1.1) |  |  |  |
|  | Missing | 3 | 3 | 0 |  |
| ***Father* employment** | Full time | 66 (33.5) | 55 (83.3) | 11 (16.7) | Ref |
|  | Part time/ Casual | 21 (10.7) | 19 (90.5) | 2 (9.5) | 0.43 |
|  | unemployed | 78 (39.5) | 73 (93.6) | 5(6.4) | 0.62 |
|  | Unknown | 32 (16.2) | 29 (90.6) | 3 (9.4) | 0.98 |
|  | Missing | 3 | 3 |  |  |
| ***Mother* employment** | Full time | 12 (6.1) | 7 (58.3) | 5 (41.7) | Ref |
|  | Part time/ Casual | 15 (7.6) | 13(86.7) | 2 (13.3) | 0.007 |
|  | Unemployed | 167 (84.8) | 153 (91.6) | 14 (8.4) | 0.003 |
|  | Unknown | 3 (1.5) | 3(1.7) | 0 |  |
|  | Missing | 3 |  |  |  |
| **Annual household income** | >78,000 | 19 (9.5) | 15 (78.9) | 4 (21.1) | Ref |
|  | $52 000-78,000 | 26 (13.0) | 22 (84.6) | 4 (15.3) | 0.31 |
|  | 26,000 - < $52 000 | 74 (37.0) | 68 (91.8) | 6 (8.1) | 0.11 |
|  | < 26,000 | 79 (39.5) | 72 (91.1) | 7 (38.9) | 0.14 |
|  | Missing | 2 | 2 | 0 |  |
| **Health insurance** | No | 186 (94.9) | 168 (90.3) | 18 (9.7) | Ref |
|  | Yes | 10 | 7(70.0) | 3(30.0) | 0.07 |
|  | Missing | 4 | 4 |  |  |

**Supplementary Table 3. Household characteristics of children who developed recurrent ARIwC**

|  |  | All children  N= 200(%) | Recurrent ARI No  n=179 (%) | Recurrent ARI Yes  n = 21 (%) | P  value |
| --- | --- | --- | --- | --- | --- |
| **Care type at home** | Both Parents | 105 (52.5) | 92 (87.6) | 13 (12.4) | Ref |
|  | Single Parents | 83 (41.5) | 75 (90.3) | 8 (9.7) | 0.39 |
|  | Other care | 12 (6.0) | 12 (6.7) | 0 |  |
| **Maternal alcohol use pre pregnancy** | No | 94 (47.7) | 83 (88.3) | 11 (11.7) | Ref |
|  | Yes | 103 (52.3) | 93 (90.3) | 10 (9.7) | 0.65 |
|  | Missing | 3 | 3 | 0 |  |
| **Maternal ETS* exposure during pregnancy** | No | 70 (35.5) | 61(87.1) | 9 (12.9) | Ref |
|  | Yes | 127 (64.5) | 115 (90.5) | 12 (9.5) | 0.47 |
|  | Missing | 3 | 3 | 0 |  |
| **Maternal smoking during pregnancy** | No | 99 (50.2) | 87 (87.9) | 12 (12.1) | Ref |
|  | Yes | 98 (49.8) | 89 (90.8) | 9 (9.2) | 0.50 |
|  | Missing | 3 | 3 | 0 |  |
| **Child exposed to ETS** | No | 54 (27.4) | 47 (87.0) | 7 (13.0) | Ref |
|  | Yes | 143 (72.6) | 129 (90.2) | 14 (9.8) | 0.52 |
|  | Missing | 3 | 3 | 0 |  |
| **Childcare attendance** | No | 140 (71.1) | 127 (90.7) | 13 (9.3) | Ref |
|  | Yes | 57 (28.9) | 49 (86.0) | 8 (14.0) | 0.32 |
|  | Missing | 3 | 3 | 0 |  |
| **Pets** | No | 87 (44.1) | 77 (88.5) | 10 (11.5) | Ref |
|  | Yes | 110 (55.9) | 99 (90.0) | 11 (10.0) | 0.78 |
|  | Missing | 3 | 3 (1.7) | 0 |  |
| **Mould in house** | No | 131 (65.5) | 112 (85.5) | 19 (14.5) | Ref |
|  | Yes | 66 (33.5) | 64 (97.0) | 2 (3.0) | 0.009 |
|  | Missing | 3 | 3 | 0 |  |
| **Damp in house** | No | 145 (73.6) | 125 (86.2) | 20 (13.8) | Ref |
|  | Yes | 52 (26.4) | 51(98.0) | 1(2.0) | 0.01 |
|  | Missing | 3 |  | 0 |  |
| **Smell in house** | No | 155 (78.7) | 135 (87.1) | 20 (12.9) | Ref |
|  | Yes | 42 (21.3) | 41 (97.6) | 1(2.4) | 0.05 |
|  | Missing | 3 |  | 0 |  |
| **House condition** | Excellent | 33 (16.8) | 27 (81.8) | 6 (18.2) | Ref |
|  | Good/Average | 146 (74.1) | 131 (89.7) | 15 (10.3) | 0.20 |
|  | Poor/very poor | 18 (9.1) | 18 (10.2) | 0 |  |
|  | Missing | 3 | 3 | 0 |  |
| **Other children in household** | None | 39 (19.8) | 36 (92.3) | 3(7.7) | Ref |
|  | 1-2 children | 107 (54.3) | 95 (88.8) | 12(11.2) | 0.53 |
|  | 3 + children | 51 (25.9) | 45 (88.2) | 6 (11.8) | 0.52 |
|  | Missing | 3 | 3 | 0 |  |
| **Total number of people in house** | 2 | 10 (5.1) | 9 (90.0) | 1 (10.0) | Ref |
|  | 3-4 | 88 (44.7) | 77 (87.5) | 11 (12.5) | 0.82 |
|  | 5-6 | 75 (38.0) | 68 (90.7) | 7 (9.3) | 0.94 |
|  | 7+ | 24 (12.2) | 22 (91.7) | 2(8.3) | 0.87 |
|  | Missing | 3 | 3 | 0 |  |

|  |  | All children  N = 180 (%) | Recurrent ARI No  n = 164 (%) | Recurrent ARI Yes  n = 16(%) | P value |
| --- | --- | --- | --- | --- | --- |
| **Cultural connection at home** | No | 68 (37.7) | 64 (94.1) | 4 (5.9) | Ref |
|  | Yes | 110 (61.1) | 98 (89.1) | 12 (10.9) | 0.26 |
|  | Unknown | 2 (1.1) | 2 (1.2) | 0 |  |
| **Family from stolen generation** | No | 42 (23.3) | 39 (92.9) | 3 (7.1) | Ref |
|  | Yes | 82 (45.6) | 74 (90.2) | 8 (9.8) | 0.62 |
|  | Unknown | 56 (31.1) | 51 (91.1) | 5 (8.9) | 0.75 |
| **Connection to country** | No | 93 (51.7) | 85(91.4) | 8 (8.6) | Ref |
|  | Yes | 78 (43.3) | 70 (89.7) | 8 (10.3) | 0.58 |
|  | Unknown | 9 (5.0) | 9 (5.0) | 0 |  |
| **Identify with Mob/Nation** | No | 77 (42.8) | 70 (90.9) | 7 (9.1) | Ref |
|  | Yes | 103 (57.2) | 94 (91.3) | 9 (8.7) | 0.93 |

**Supplementary Tables 4: Cultural characteristics of Indigenous children who developed recurrent ARI**

**Supplementary Table 5: Child characteristics of children who developed chronic cough**

|  |  | All children  N=200 (%) | Chronic cough N  n = 157 | Chronic cough Y  n = 43 (%) | ***P*** value |
| --- | --- | --- | --- | --- | --- |
| **Gender** | Female | 95 (47.5) | 72 (75.8) | 23 (24.2) | Ref |
|  | Male | 105 (52.5) | 85 (80.9) | 20 (19.1) | 0.37 |
| **Indigenous Status** | Non-Indigenous | 20 (10.0) | 12 (60.0) | 8 (40.0) | Ref |
|  | Indigenous | 180 (90.0) | 145 (80.6) | 35 (19.4) | 0.03 |
| **Age group months** | 36 ->60* | 46 (23.0) | 40 (86.0) | 6 (13.0) | Ref |
|  | <24 - <36 months | 32 (16.0) | 30 (93.7) | 2(6.3) | 0.34 |
|  | > 12 - <24 months | 55 (27.5) | 39 (70.9) | 16 (29.1) | 0.05 |
|  | 6 -<12 months | 30 (15.0) | 21 (70.0) | 9 (30.0) | 0.07 |
|  | < 6 months | 37 (18.5) | 27 (73.0) | 10 (27.0) | 0.11 |
| **Birth Weight (grams)** | ≥2500 | 161 (80.5) | 127 (78.9) | 34 (21.1) | Ref |
|  | ≤ 2500 | 39 (19.5) | 30 (76.9) | 9 (23.1) | 0.78 |
| **Gestational age weeks** | ≥ 37 | 159 (79.5) | 126 (79.2) | 33 (20.8) | Ref |
|  | < 37 | 41 (21.0) | 31 (75.6) | 10 (24.4) | 0.61 |
| **History of wheeze past 12 months** | No | 123 (61.0) | 99 (80.5) | 24 (19.5) | Ref |
|  | Yes | 75 (38.0) | 56 (74.7) | 19 (25.3) | 0.03 |
|  | Missing | 2 | 2 | 0 |  |
| **History of Eczema ever** | No | 174 (87.9) | 139 (79.9) | 35 (20.1) | Ref |
|  | Yes | 24 (12.1) | 16 (66.7) | 8 (33.3) | 0.14 |
|  | Missing | 2 | 2 | 0 |  |
| **Hospitalised for ARI 12 months prior to enrolment** | No | 173 (87.3) | 135 (78.0) | 38 (22.0) | Ref |
|  | Yes | 25 (12.7) | 20 (80.0) | 5 (20.0) | 0.82 |
|  | Missing | 2 | 2 | 0 |  |
| **Cough> 4 weeks duration** **12 months prior to enrolment** | No | 151 (76.3) | 124 (82.1) | 27 (17.9) | Ref |
|  | Yes | 47 (23.7) | 31 (66.0) | 16 (34.0) | 0.02 |
|  | Missing | 2 | 2 | 0 |  |
| **Respiratory Diagnosis 12 months prior to enrolment** | No | 130 (65.6) | 106 (81.5) | 24 (18.5) | Ref |
|  | Yes | 68 (34.3) | 49 (72.1) | 19 (27.9) | 0.12 |
|  | Missing | 2 | 2 | 0 |  |
| **Season enrolled** | Summer | 57 (28.5) | 48 (84.2) | 9 (15.8) | Ref |
|  | Autumn | 53 (26.5) | 42 (79.2) | 11 (20.8) | 0.50 |
|  | Winter | 37 (18.5) | 31 (83.8) | 6 (16.2) | 0.95 |
|  | Spring | 53 (26.5) | 36 (67.9) | 17 (32.1) | 0.04 |

**Supplmentary Table 6. Parental characteristics of children who developed chronic cough**

| **Maternal age at birth of child** | 40 + years | 5 (2.8) | 4 (10.8) | 1(25.6) | Ref |
| --- | --- | --- | --- | --- | --- |
|  | 35 – <40 years | 15 (8.4) | 9 (16.6) | 6 (14.0) | 0.83 |
|  | 30 – <35 years | 29 (16.2) | 45 (18.4) | 4 (16.2) | 0.92 |
|  | 25 - <30 years | 37 (20.7) | 29 (52.9) | 8 (44.2) | 0.93 |
|  | <25 years | 93 (51.9) | 77 (1.3) | 16 |  |
|  | Missing | 2 |  |  |  |
| **Paternal age at birth of child** | 40+ years | 21(10.7) | 14 (22.2) | 7 (34.9) | Ref |
|  | 35 – <40 years | 28(14.2) | 20 (12.8) | 8 (9.3 ) | 0.72 |
|  | 30 – <35 years | 25 (12.7) | 20 (26.7) | 2 (27.9) | 0.17 |
|  | 25 - <30 years | 53 (26.9) | 41 (37.0) | 12 (27.9) | 0.34 |
|  | < 25 years | 70(35.5) | 58 | 12 | 0.11 |
|  | Missing | 3 | 1 |  |  |
| **Indigenous Parent status** | Both Indigenous | 59 (30.0) | 53(89.9) | 6 (10.1) | Ref |
|  | Indigenous dad/ non-Indigenous mum | 59 (30.0) | 47(79.7) | 12(20.3) | 0.13 |
|  | Indigenous mum/ non-Indigenous dad | 59 (30.0) | 42(71.2) | 17(28.8) | 0.01 |
|  | Both non-Indigenous | 20(10.0) | 12 (60.0) | 8 (40.0) | 0.005 |
| **Father education** | Tertiary degree | 2 (1.0) | 2 (1.3) | 0 | Ref |
|  | Secondary school/ trade | 82 (41.8) | 58 (70.3) | 24 (29.3) | 0.99 |
|  | Did not complete high school | 82 (41.8) | 68 (82.9) | 14 (17.1) | 0.99 |
|  | Unknown | 30 (15.4) | 25 (83.3) | 5(16.7) | 0.99 |
|  | Missing | 4 | 4 | 0 |  |
| **Mother education** | Tertiary degree | 10 (5.0) | 4 (40.0) | 6 (60.0) | Ref |
|  | Secondary school/ trade | 108 (54.9) | 82 (75.9) | 26 (24.1) | 0.02 |
|  | Did not complete high school | 77 (39.1) | 66 (85.7) | 11 (14.3) | 0.002 |
|  | Unknown | 2(1.0) | 2 (1.3) |  |  |
|  | Missing | 3 |  |  |  |
| **Father employment** | Full time | 66 (33.5) | 46 (69.7) | 20 (30.3) | Ref |
|  | Part time/ Casual | 21 (10.7) | 16 (76.2) | 5 (23.8) | 0.56 |
|  | unemployed | 78 (39.6) | 64 (82.1) | 14 (17.9) | 0.54 |
|  | Unknown | 32(16.2) | 28(87.5) | 4(12.5) | 0.29 |
|  | Missing | 3 | 3 |  |  |
| **Mother employment** | Full time | 12 (6.1) | 8 (66.7) | 4 (33.3) | Ref |
|  | Part time/ Casual | 15 (7.6) | 10 (66.7) | 5 (33.3) | 1.0 |
|  | Unemployed | 167 (84.8) | 133 (79.6) | 34 (20.4) | 0.24 |
|  | Unknown | 3(1.5) | 3 (1.95 |  |  |
|  | Missing | 3 | 3 |  |  |
| **Annual household income** | >78,000 | 19 (9.5) | 10 (52.6) | 9 (47.4) | Ref |
|  | $52 000-78,000 | 26 (13.0) | 20 (76.9) | 6 (23.1) | 0.09 |
|  | 26,000 - < $52 000 | 74 (37.0) | 64 (86.5) | 10 (13.5) | 0.002 |
|  | < 26,000 | 79 (39.5) | 61 (77.2) | 18 (22.8) | 0.03 |
|  | Missing | 2 (1.0) | 2 (1.2) |  |  |
| **Health insurance** | No | 186 (93.0) | 148 (79.6) | 38 (20.4) | Ref |
|  | Yes | 10 (5.0) | 5 (50.0) | 5 (50.0) | 0.04 |
|  | Missing | 4 | 4 | 0 |  |
| **Primary carer on welfare** | No | 19 (9.6) | 17 (89.5) | 2 (10.5) | Ref |
|  | Yes | 180 (90.4) | 139 (77.2) | 41 (22.8) | 0.21 |
|  | Missing | 1 | 1 | 0 |  |

**Supplmentary Table .7: Table x. Household characteristics of children with chronic cough**

|  |  | All children  N= 200  n (%) | Chronic Cough  No  n=157 (%) | Chronic Cough  Yes  n = 43 (%) | P  value |
| --- | --- | --- | --- | --- | --- |
| **Care type at home** | Both Parents | 105 (52.5) | 81 (77.1) | 24 (22.9) | Ref |
|  | Single Parents | 83 (41.5) | 67 (80.7) | 16 (19.3) | 0.80 |
|  | Other care | 12 (6.0) | 9 (75.0) | 3 (25.0) |  |
| **Maternal alcohol use pre pregnancy** | No | 94 (47.0) | 75 (79.8) | 19 (20.2) | Ref |
|  | Yes | 103 (51.5) | 79 (76.7) | 24 (23.3) | 0.60 |
|  | Missing | 3 | 3 | 0 |  |
| **Maternal ETS* exposure during pregnancy** | No | 70 (35.0) | 54 (77.1) | 16 (22.9) | Ref |
|  | Yes | 127 (63.5) | 100 (78.7) | 27 (21.3) | 0.79 |
|  | Missing | 3 | 3 | 0 |  |
| **Maternal smoking during pregnancy** | No | 99 (49.5) | 73 (73.7) | 26 (26.3) | Ref |
|  | Yes | 98 (49.0) | 81 (82.7) | 17 (17.3) | 0.16 |
|  | Missing | 3 | 3 | 0 |  |
| **Child exposed to ETS** | No | 54 (27.0) | 39 (72.2) | 15 (27.8) | Ref |
|  | Yes | 143 (71.5) | 115 (80.4) | 28 (19.6) | 0.21 |
|  | Missing | 3 | 3 | 0 |  |
| **Childcare attendance** | No | 140 (70.0) | 114 (81.4) | 26 (18.6) | Ref |
|  | Yes | 57 (28.5) | 40 (70.2) | 17 (29.8) | 0.08 |
|  | Missing | 3 | 3 | 0 |  |
| **Pets** | No | 87 (43.5) | 72(82.8) | 15 (17.2) | Ref |
|  | Yes | 110 (55.0) | 82 (74.5) | 28 (25.5) | 0.25 |
|  | Missing | 3 | 3 | 0 |  |
| **Mould in house** | No | 131 (65.5) | 100 (76.3) | 31 (23.6) | Ref |
|  | Yes | 66 (33.0) | 54 (81.8) | 12 (18.2) | 0.37 |
|  | Missing | 3 | 3 | 0 |  |
| **Damp in house** | No | 145 (72.5) | 113 (77.9) | 32 (22.1) | Ref |
|  | Yes | 52 (26.0) | 41 (78.8) | 11 (21.2) | 0.89 |
|  | Missing | 3 | 3 | 0 |  |
| **Smell in house** | No | 155 (78.6) | 121 (78.1) | 34(21.9) | Ref |
|  | Yes | 42 (21.0) | 33(78.6) | 9 (21.4) | 0.94 |
|  | Missing | 3 | 3 | 0 |  |
| **House condition** | Excellent | 33 (16.5) | 25 (75.8) | 8 (24.2) | Ref |
|  | Good/Average | 146 (73.0) | 112 (76.7) | 34 (23.3) | 0.90 |
|  | Poor/very poor | 18 (9.0) | 17(94.4) | 1 (5.6) | 0.12 |
|  | Missing | 3 | 3 | 0 |  |
| **Other children in household** | None | 39 (19.5) | 30 (76.9) | 9 (23.1) | Ref |
|  | 1-2 children | 107 (53.5) | 84(78.5) | 23 (21.5) | 0.97 |
|  | 3 + children | 51 (25.5) | 40 (78.4) | 11(21.6) | 0.86 |
|  | Missing | 3 | 3 | 0 |  |
| **Total number of people in house** | 2 | 10 (5) | 9 (90.0) | 1 (10.0) | Ref |
|  | 3-4 | 88 (44) | 69 (78.4) | 19 (21.6) | 0.40 |
|  | 5-6 | 75 (37.5) | 57 (76.0) | 18 (24.0) | 0.33 |
|  | 7+ | 24 (12) | 19 (79.2) | 5 (20.8) | 0.46 |
|  | Missing | 3 | 3 | 0 |  |

**Supplementary Table 8: Cultural characteristics of Indigenous children with chronic cough**

|  |  | All children  N = 180  (%) | Chronic Cough No  n = 145  n (%) | Chronic Cough Yes  n = 35  n (%) | P value |
| --- | --- | --- | --- | --- | --- |
| **Cultural connection at home** | No | 68 (37.8) | 58 (85.3) | 10 (14.7) | Ref |
|  | Yes | 110 (61.1) | 85 (77.3) | 25 (22.7) | 0.19 |
|  | Unknown | 2 (1.1) | 2 (1.4) | 0 | 0 |
| **Family from stolen generation** | No | 42 (23.3) | 34 (80.9) | 8 (19.1) | Ref |
|  | Yes | 82 (45.6) | 62 (75.6) | 20 (24.4) | 0.22 |
|  | Unknown | 56 (31.1) | 49(87.5) | 7 (12.5) | 0.37 |
| **Connection to country** | No | 93 (51.7) | 78 (83.9) | 15 (16.1) | Ref |
|  | Yes | 78 (43.3) | 60 (76.9) | 18 (23.1) | 0.25 |
|  | Unknown | 9 (5.0) | 7 (77.8) | 2(22.2) | 0.64 |
| **Identify with Mob/Nation** | No | 77 (42.8) | 64 (83.1) | 13(16.9) | Ref |
|  | Yes | 103 (57.2) | 81 (78.6) | 22 (21.4) | 0.45 |
